# The power of peers

**DOI:** 10.1080/13814788.2026.2678753

**Published:** 2026-05-28

**Authors:** Jako Burgers

In both science and life, we rarely make progress on our own. From early education to our professional maturity, the presence of a strong peer group shapes the way we think, grow, and perform. In our youth, peers influence our development and well-being; in adult life, they continue to offer us support, challenges, and inspiration. The same principle applies to scientific publications. Behind every high-quality journal stands a dedicated community of peers.

## The EJGP community

For the *European Journal of General Practice* (EJGP), peer reviewers are the cornerstone of quality. Their work often goes unnoticed, is carried out quietly and without formal remuneration, yet it is of vital importance. Through their critical appraisal, constructive feedback, and professional expertise, reviewers ensure that published articles meet the scientific standards our readership expects. They ensure methodological rigour, enhance clarity, and help authors refine their work [1,2]. In doing so, they contribute not only to individual manuscripts but also to the advancement of general practice as a specialty and to strengthening the journal’s credibility and impact.

It is important to note that our reviewers do not form a detached or isolated group. They are part of a vibrant, interconnected community. Many of them are authors who publish in EJGP themselves, and readers who apply the findings in their own research and practice. This dual role strengthens the journal: reviewers understand the challenges of authorship, and those who publish appreciate the value of a thoughtful peer review. It is this reciprocal relationship that gives the journal its strength and sustainability and fosters a shared sense of commitment and connection.

## Addressing the challenges

The landscape of academic publishing has changed considerably over the past decade. The number of journals has grown rapidly, and competition has increased not only for high-quality submissions but also for reviewers. Although peer review is essential for a high-quality scientific journal, reviewers’ work is largely carried out on a voluntary basis. As a result, editors across a wide range of disciplines are finding it increasingly difficult to obtain timely and suitable reviews. This can lead to delays in the editorial process and place additional strain on a limited group of dedicated reviewers [3].

Given these challenges, we recently undertook an initiative to strengthen our reviewer community. We reached out to colleagues within our International Group of Advisors and the European General Practice Research Network (EGPRN), inviting them to indicate their willingness to review manuscripts for EJGP and to specify their areas of expertise. The response exceeded our expectations. Not only was participation high, but the level of enthusiasm and commitment to the journal was deeply encouraging.

Based on this initiative, we have compiled a list of 164 potential reviewers from 46 countries covering a broad range of topics relevant to general practice and primary care research. Diversity in geographical background broadens the range of perspectives and increases the likelihood that contextual limitations are identified. The resource will support our editorial process by facilitating more efficient matching of manuscripts to reviewers, thereby improving both the speed and quality of peer review. Just as importantly, it reinforces the sense that EJGP is supported by an internationally engaged and responsive community of scholars.

## The EJGP Best Paper Award 2025

While acknowledging the importance of peer review, we must also celebrate the outcomes it helps to achieve. A thorough review contributes directly to high-quality papers, and it is therefore justified to recognise outstanding articles among those published. We are delighted to announce the EJGP Best Paper Award 2025, a prize awarded to outstanding research that demonstrates methodological rigour, relevance to practice, and contribution to the field.

The best paper has been selected from 31 original research articles published in our journal in 2025. The winner is ‘*Identifying opportunities for shared decision-making through patients’ and physicians’ perceptions on the diagnostic process: A qualitative analysis of malpractice claims in general practice*’, written by Sophie Jacobse and colleagues [4]. By examining real-world cases, the paper offers valuable insights into communication gaps, clinical reasoning, and the complexity of diagnostic interactions in general practice. It highlights how greater patient involvement could improve diagnostic safety and reduce misunderstandings in healthcare. We concluded that the article is outstanding in terms of its relevance to general practice and its potential impact on improving patient-centered care. The clarity, relevance, and academic rigour demonstrated in this study set the bar high for future research. Congratulations!

## Building on a strong foundation

Looking ahead, the continued success of EJGP depends on the active involvement of its community. We encourage colleagues to contribute as reviewers and share their expertise to support the next generation of researchers. We invite authors to submit their best work, confident that it will be assessed carefully and fairly. We ask readers to critically evaluate the articles we publish, apply the findings, and cite them in future research. In this way, every member of the community contributes to strengthening the journal.

The power of peers lies not only in their individual contributions but also in their collective efforts. Our journal is more than just a platform for publications; it is a network of relationships, a space for dialogue, and a shared commitment to the advancement of knowledge. By supporting one another—as reviewers, authors, and readers—we ensure that EJGP continues to flourish as a reliable source of high-quality research in the field of general practice.

Ultimately, the strength of a journal reflects the strength of the community behind it. We are grateful for the commitment of our peer reviewers and look forward with optimism to what we can achieve together, both now and in the future.


Jako Burgers *Editor-in-Chief*
jako.burgers@maastrichtuniversity.nl


